# An integrated analysis of membrane remodeling during porcine reproductive and respiratory syndrome virus replication and assembly

**DOI:** 10.1371/journal.pone.0200919

**Published:** 2018-07-24

**Authors:** Wei Zhang, Keren Chen, Xueqing Zhang, Chunhe Guo, Yaosheng Chen, Xiaohong Liu

**Affiliations:** 1 School of Life Sciences, Sun Yat-Sen University, Guangzhou, P. R. China; 2 State Key Laboratory of Biocontrol, School of Life Sciences, Sun Yat-Sen University, Guangzhou, P. R. China; South China Agricultural University, CHINA

## Abstract

**Background:**

Recently, three-dimensional (3D) imaging techniques have been used to detect viral invasion and the appearance of specialized structures established in virus-infected cells. These methods have had a positive impact in the field of virology and helped to further our knowledge of how viruses invade cells. Nearly all positive-strand RNA viruses propagate their viral genomes in part through intracellular membranes. Porcine reproductive and respiratory syndrome virus (PRRSV), an Arterivirus, accumulates viral RNA that forms replication complexes (RCs) in infected cells. In this study, using immunofluorescence and electron microscopy (EM), we dissected PRRSV-induced membrane structures in infected cells and determined the correlations between PRRSV particles and vesicles stimulated by PRRSV to understand the structural and dynamic aspects of PRRSV infection.

**Methods:**

We identified the appropriate time point by determining the 50% tissue culture infectious dose (TCID50) and using qRT-PCR and Western blotting. The co-localization of viruses and organelles was determined by immunofluorescence and immune-electron microscopy (IEM). The ultrastructure of cells infected by PRRSV was observed using EM and electron tomography (ET).

**Results:**

In our study, we found that PRRSV dsRNA was located at the endoplasmic reticulum (ER) and autophagosomes; in addition, the N protein was located at the mitochondria, ER and autophagosomes. Vesicles induced by PRRSV appeared at 16 hours post-infection (h.p.i.) and increased in size with time during the infection period. In addition, our findings demonstrated that the virus vesicles originated from the ER, and these two organelle structures connected with each other to form a reticulovesicular network (RVN) that provided a site for virus replication and assembly.

**Conclusion:**

Our results revealed that membrane vesicles induced by PRRSV were derived from the ER. The vesicles may provide a location for PRRSV replication and assembly.

## Introduction

Porcine reproductive and respiratory syndrome virus (PRRSV), whose virions are 50–65 nm in diameter, belongs to the order *Nidovirales* and the family *Arteriviridae*, which includes equine arteritis virus (EAV), lactate dehydrogenase-elevating virus (LDV), and simian haemorrhagic fever virus (SHFV) [1]. PRRSV is a small, enveloped, positive-strand RNA viral pathogen that encodes a polycistronic RNA genome consisting of a 15-kb long 3'-polyadenylation molecule that is responsible for detrimental reproductive disturbance, post-weaning pneumonia, growth disorders, low physiological performance, and a notable death rate in the swine industry. The first outbreaks of PRRSV were reported in Germany in 1990 and were far-flung throughout Europe by 1991 [2]. The outbreak of PRRSV in China in 2006 received global attention [3]. To date, PPRSV continues to spread and cause severe economic losses in the porcine industry worldwide.

After PRRSV infects a cell, the internal cellular morphology and structure change. Dramatic membrane rearrangements, which support viral replication and assembly, often occur in host cells upon virus invasion [4]. Widespread virus-induced membrane remodelling is the most prominent morphological feature observed in images of PRRSV-infected cells. This phenomenon was described more than 20 years ago, and the shapes, properties and formation mechanisms of these replicative structures have been characterized [5]. It has been shown that PRRSV mRNA is translated into a single polyprotein associated with certain organelles, such as mitochondria and the endoplasmic reticulum (ER), and these structures are morphologically similar to autophagosomes [6, 7]. Synchronously, the polyprotein is cleaved by cellular and viral proteases, which produce individual proteins required for later viral RNA synthesis and virion assembly. Furthermore, several studies have identified extensive cytoskeletal alterations induced by various members of the order *Nidovirales*. For instance, microtubule networks and double-membrane vesicles (DMVs) are reorganized in cells infected with EAV, and the innate immune system interfaces with the formation of replication organelles [8–12].

Some positive-strand RNA (+RNA) viruses induce membranous vesicles (MVs) to assemble virus replication complexes (RC) in infected cells. To a certain extent, viral RNA synthesis and the formation of replication-transcription complexes (RTCs) benefit from induced MVs. It is believed that viral proteins, RNA and some factors from the host cell create a scaffold for RCs, favouring viral RNA synthesis [12]. Additionally, membranes induced by the virus assist viral RNA, especially double-stranded RNA (dsRNA), with escaping the host immune response to replicate [13]. Different viruses form their RCs on specific cellular organelles, such as the outer mitochondrial membrane, the ER [14–16], the Golgi complex [16, 17] or the plasma membrane [15, 18]. Benefitting from electron microscopy (EM) and electron tomography (ET) technology, many studies have described the membrane rearrangements induced by positive-strand RNA viruses, including nodavirus (flock house virus), flaviviruses (dengue, West Nile and hepatitis C virus) and togavirus (rubella virus). Of note, in studies of other members of *Nidovirales*, the replication of EAV and three coronaviruses, specifically mouse hepatitis virus (MHV) [19], severe acute respiratory syndrome coronavirus (SARS) [20] and Middle East respiratory syndrome coronavirus (MERS-CoV), induced membrane rearrangement, including DMVs and reticular inclusions or complex membranes [20, 21]. DMVs were also observed during infection with infectious bronchitis virus (IBV), another coronavirus, and a small proportion of these DMVs were connected to the ER through their outer membrane, forming a zipped ER region [22].

For PRRSV infection, membrane rearrangements are established when highly heterogeneous MVs accumulate in the cytoplasmic matrix [23]. In addition, nidoviruses such as corona- and arteriviruses induce reticulovesicular networks (RVNs) of interconnected ER-derived DMVs and other membrane rearrangements. The RVN is thought to provide a location for viral replication or assembly and to protect the RC from innate immune detection [8]. However, in PRRSV-infected cells, the three-dimensional architecture of the membrane network, its occurrence, and the site of RNA replication have not yet been elucidated. A study was conducted in the 1990s to describe host cell modifications during PRRSV replication, but no other in-depth research has been conducted to identify the particular role of PRRSV virions in cell membrane rearrangement [5]. Here, we elucidated viral RNA, protein synthesis and particle release sites and described the details of PRRSV-induced membrane rearrangement. Finally, we identified potential sites for the synthesis of viral RNA that may be released.

In this study, we combined confocal microscopy and immune-electron microscopy (IEM) with live cell imaging approaches to detect viral RNA and proteins and to identify the replication and synthesis compartments in PRRSV-infected cells. Moreover, thin-section transmission EM and ET allowed us to conveniently observe the three-dimensional (3D) structure and biogenesis of PRRSV-induced cellular membrane compartments in Marc-145 cells. In summary, this study explained the topological relationship between PRRSV infection and cellular membrane rearrangement.

## Materials and methods

### Cell culture and virus infection

Marc-145 (African green monkey kidney) cells were purchased fromAmerican Tissue Culture Collection (ATCC). Cells were cultivated in Dulbecco’s minimal essential medium (DMEM, Invitrogen, the United States) supplemented with 10% foetal calf serum (FCS) at 37°C in 5% CO_2_. Marc-145 cells were infected with PRRSV strain CH-1a (the first type 2 PRRSV strain extracted and isolated in South China, offered by Dr. Guihong Zhang from South China Agricultural University, China). According to the Reed Muench method, a 50% tissue culture infectious dose was counted every hour post-infection (h.p.i.).

### Antibodies

Mouse anti-PRRSV N protein and monoclonal J2 anti-dsRNA antibodies were purchased from Jeno Biotech Inc. (Chuncheon, South Korea) and Scicons (Hungary), respectively. Cellular DNA, mitochondria and lysosomes were stained with DAPI, MitoTracker Red and LysoTracker Red, respectively (all reagents were from Molecular Probes, OR, USA). Rabbit anti-calnexin, anti-LC3 and anti-IgG Alexa Fluor were purchased from Cell Signaling Technology. A goat anti-mouse IgG-Gold antibody was obtained from Sigma-Aldrich.

### qRT-PCR

Cells were washed with phosphate-buffered saline (PBS) and collected. Total RNA was extracted using TRIzol™ reagent (Invitrogen, United States), and a reverse transcription kit (Promega, Madison, WI, USA) was used to synthesize complementary DNA (cDNA). After transient centrifugation, a quantitative plate mixture was placed in a LightCycler® 480 (Roche) according to real-time PCR system protocols [24]. The reaction (total volume of 10 μL) contained 5 μL of Ex Taq™ (TaKaRa, China), 1 μL of cDNA template (500 ng/mL), 3.4 μL of sterile distilled water, and 0.3 mL of forward and reverse primers, each at a concentration of 10 μmol/L. The reaction process consisted of 95°C for 10 seconds, followed by 40 cycles of 95°C for 10 seconds and 60°C for 30 seconds, and finally 72°C for 30 seconds. Specific primer sequences for the viral N genes were found in the NCBI database: N-F, 5′-AAAACCAGTCCAGAGGCAAG-3′; and N-R, 5′-CGGATCAGACGCACAGTATG-3′.

### Western blotting

Cells were washed and then added into cell lysis buffer (Beyotime Biotechnol, Shanghai, China) with 1 mM diethyl pyrocarbonate (PMSF). Sample proteins were extracted with lab-made protein lysate and heated at 80°C for 5 minutes, followed by 12% sodium dodecyl sulphate polyacrylamide gel electrophoresis (SDS-PAGE). When electrophoresis was complete, proteins in the 12% gel were blotted onto a polyvinyl difluoride (PVDF) membrane and then sealed with 0.05% TBS Tween supplemented with 3% FCS for at least 1 h, followed by incubation of the PVDF membrane with a specific primary antibody overnight at 4°C. Eight to twelve hours later, the PVDF membrane was washed three times, 10 minutes per wash, and then incubated with an HRP-conjugated secondary antibody at room temperature for 1 hour. An image was obtained using chemiluminescence substrate (Pierce, IL, USA) and an Image Station 4000 mm PRO system (Kodak).

### Confocal microscopy

Cells that detached from virus-infected plates were seeded onto glass coverslips, followed by three PBS washes and paraformaldehyde (PFA) fixation for 15 minutes at room temperature. The cells were then permeabilized with 0.5% Triton X 100 for 5 minutes and sealed in PBS buffer supplemented with 1% bovine serum albumin (BSA) for 1 hour at normal temperature. Later, a primary antibody was added onto the abovementioned coverslips, which were incubated overnight in PBS buffer with 1% BSA at 4°C. On the second day, the cells were washed with PBS three times (5 minutes each time) and incubated with a primary antibody that was dissolved in PBS/1% BSA for 1 hour at room temperature. After three PBS washes, the cells were mixed with an Alexa Fluor-conjugated secondary antibody (diluted 1:1,000) for 1 hour at room temperature in the dark. After an additional 3 PBS washes, cellular nuclei were stained with Hoechst dye 33258 (Sigma) in the dark at room temperature, followed by three PBS washes. The cells were then anchored onto microscope slides and observed using a Leica TCS SP5 confocal microscope after dye incubation for 4 minutes. Finally, an EM CCD camera was utilized with excitation at 405 nm for DAPI, 488 nm for Alexa Fluor 488, and 568 nm for Alexa Fluor 568, MitoTracker Red and LysoTracker Red, and the channels were recorded. Confocal images were captured using the ImageJ software package.

### EM and ET

Cells infected for 24 h were collected and resuspended and then centrifuged at 1000 g for 5 minutes. The cells were then fixed with 2% paraformaldehyde and 3% glutaraldehyde for 2 hours and 1% osmic acid for 90 minutes. Harvested cells were dehydrated with an ethanol gradient and wrapped in Spur resin [25]. Thereafter, thin slices (100-nm-thick) were created with an LKB2188 microtome, placed on 200-mesh carbon-coated copper grids, and stained with 2% uranyl acetate for 30 minutes before incubation with Reynold’s lead citrate for 10 minutes. The samples were sequentially visualized with a JEOL JEM-1400 transmission electron microscope at an accelerating voltage of 120 kV. For ET, 300-nm-thick sections were cut, transferred to 100-mesh carbon-coated copper grids and incubated with 2% uranyl acetate for 30 minutes and Reynold’s lead citrate for 10 minutes as previously mentioned. Colloidal gold particles (15 nm in diameter) were then applied to both sides of the slices as reference markers during image alignment. ET data were captured using an FEI Tecnai F20 microscope at 200 kV as previously described [26]. A suitable region was selected from a single-axis tilt series and collected with a 4k × 4k CCD; the samples were artificially tilted at 2° increments exceeding a range of 120° (±60°). The final magnification was 11.5k, and the A/pixel angle was 19.6°. The IMOD software package was used for reconstruction, segmentation, and rendering.

### IEM

Infected cells were harvested for later IEM analysis with LR White resin. Pelleted cells were resuspended and centrifuged at 750 g for 10 minutes, followed by fixation in a mixture of 1% paraformaldehyde, 0.5% glutaraldehyde, and 0.1 M PBS (pH 7.4) for 10 minutes at 4°C. Next, the cells were incubated in another solution supplemented with 2% paraformaldehyde, 2.5% glutaraldehyde, and 0.1 M PBS (pH 7.4) for 70 minutes at 4°C, followed by three washes with 0.1 M PBS (pH 7.4) and a dehydrated gradient of 30%, 50%, 70%, and 90% ethanol at 4°C. The samples were sequentially incubated with a gradient of LR White and ethanol (30%, 70% and 100%) at -20°C for 1 hour per step. Then, the cells were infiltrated with 100% LR White at -20°C for 12 h. The resin was pretreated for polymerization with 320-nm UV irradiation at 20°C for 72 h and 25°C for 24 h. Ultrathin sections were immune-labelled with an anti-protein N antibody (1:50) or dsRNA (1:50) for 12 h at 4°C and then incubated with goat anti-mouse IgG conjugated to 10-nm gold particles as the secondary antibody (1:50). Images of the samples were captured with a JEOL JEM-1400 transmission electron microscope at an accelerating voltage of 120 kV.

## Results

### Time course measurement of PRRSV replication in Marc-145 cells

**Fig 1:**
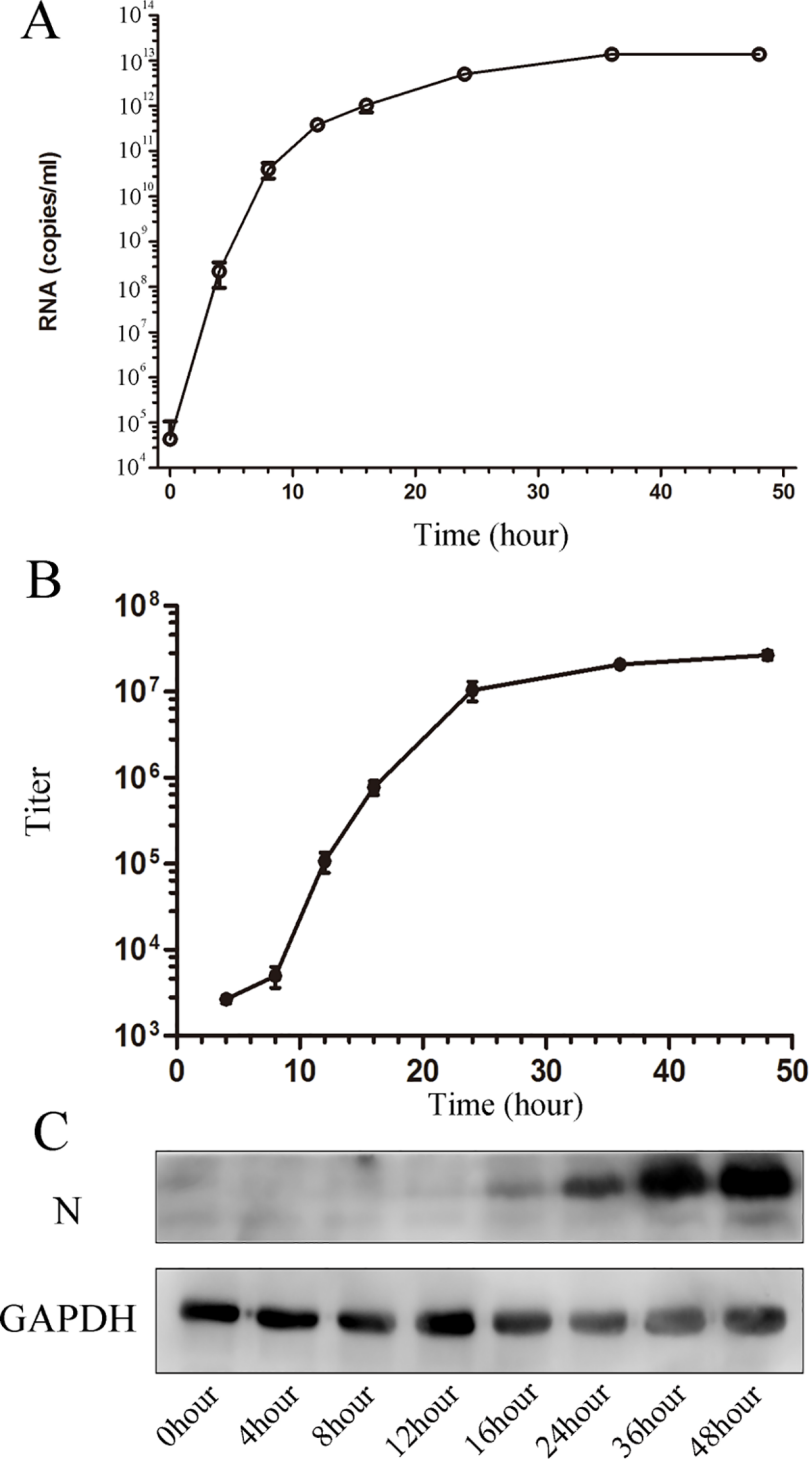
Timeline of PRRSV replication and assembly. PRRSV was transfected into Marc-145 cells at an MOI of 1and samples were obtained at different time points as mentioned. (A) Total RNA was extracted and quantified by quantitative RT-PCR through a PRRSV-specific TaqMan probe. (B) The cell-related virus titers in infected cell lysates collected at each time point were employed to analyze the extracellular virus titers. (C) Cell extractives were verified by Western blotting using anti-N antibodies; anti-GAPDH antibodies served as an internal reference.

To identify a suitable time point to analyse the infectious processes of PRRSV in Marc-145 cells, different methods were used to define the PRRSV replication rate. Marc-145 cells were infected at a multiplicity of infection (MOI) of 1. After absorption, the cells were harvested at 4, 8, 12, 16, 24, 36, and 48 h.p.i. Absolute qRT-PCR results showed that newly replicated PRRSV RNA was detectable at 4 h.p.i., and viral RNA copies increased during the exponential period until 24 h.p.i., at which point they reached a plateau (Fig 1A). Viral titres were measured to quantify infectious, cell-interacting viruses. Released progeny virus particles were observed at 12 h, which demonstrated that there was a 4-h delay in virus synthesis relative to RNA replication (Fig 1B). The nucleocapsid structural protein is dispensable for genome replication and subgenomic mRNA transcription [27]. Immunostaining results also showed that the concentration of viral N protein increased until 24 h.p.i. and then reached a plateau (Fig 1C). Virus titres and RNA levels peaked at 24 h.p.i., at which point synthesis of the viral N protein continued. Thus, the 24-h time point was the most appropriate time point for EM analysis to study the functional relationship between PRRSV-induced structures and RNA replication and identify PRRSV virus budding and membrane rearrangement.

### Membrane rearrangements induced by PRRSV infection occurred in a time-dependent manner

**Fig 2:**
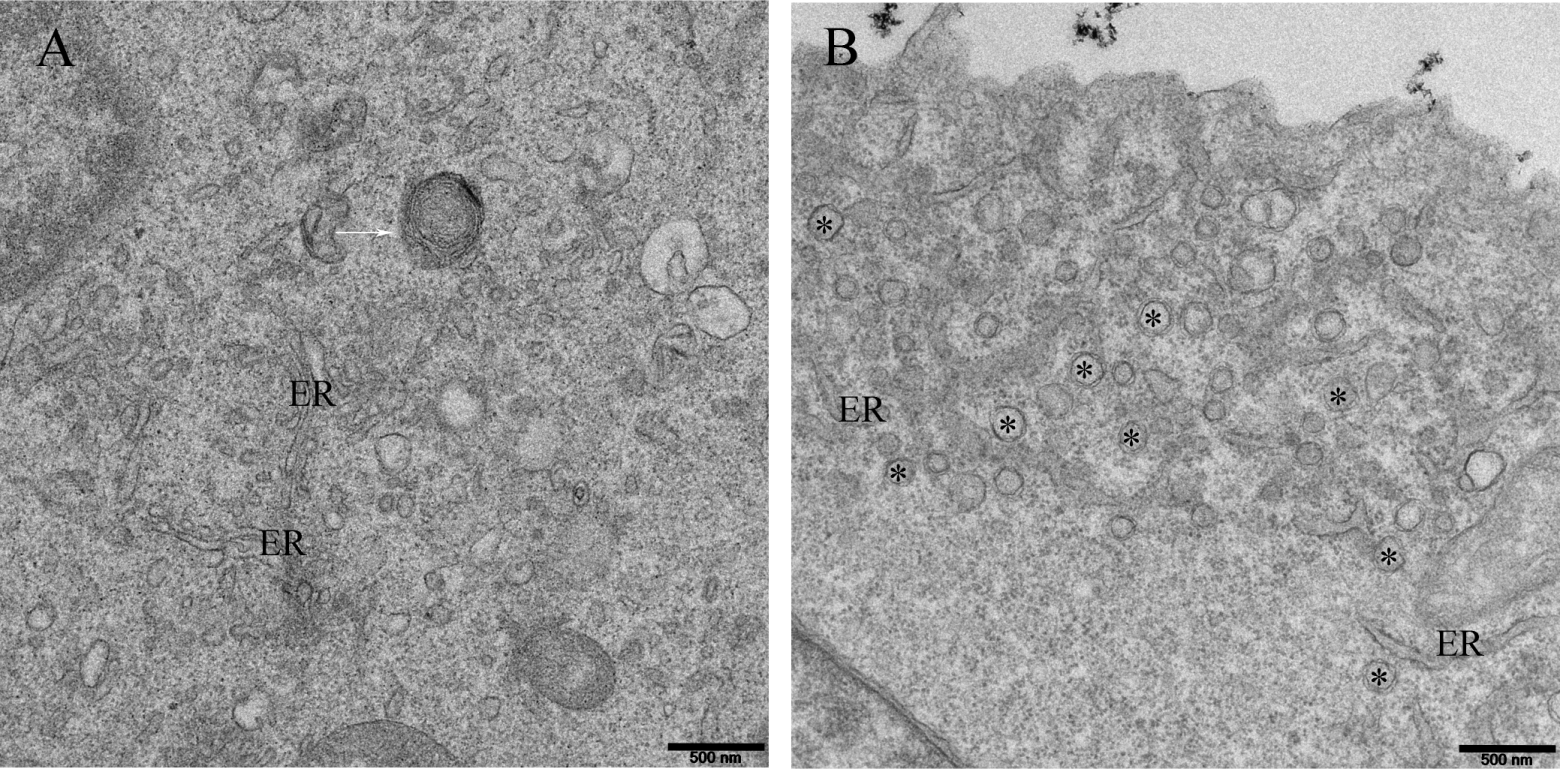
Membranous ultrastructure caused by PRRSV infection in host cells. PRRSV at an MOI of 1 was transfected into Marc-145 cell, fixed at 24 hpi, and used for conventional EM as described in the Materials and Methods. (A) A range of vesicles were detected. DMVs (✱) were labeled with asterisks; autophagosomes are annotated with white arrows. (B) DMVs are often found in clusters by a small network of membranes. Scale bars, 500 nm.

In a previous study, virus-induced DMVs were detected in cells infected with PRRSV by EM; these DMVs were most likely formed by the ER [23]. In this study, cells infected for 24 h were incubated with 4% paraformaldehyde and infused into resin. Ultrathin sections were stained, and the cellular ultrastructure was observed by EM. Fig 2A shows complex vesicular structures within the cells after 24 hours of viral invasion. Compared with mock-infected Marc-145 cells, cells infected by PRRSV appeared to contain a series of complex structures. These structures consisted of a number of vesicles, including double-layer membrane structures that were abundant in the vicinity of the ER and complex membranes and vesicles (Fig 2B). As previously reported for EAV-infected cells, a reticulovesicular network was observed in PRRSV-infected cells in addition to vesicles.

**Fig 3:**
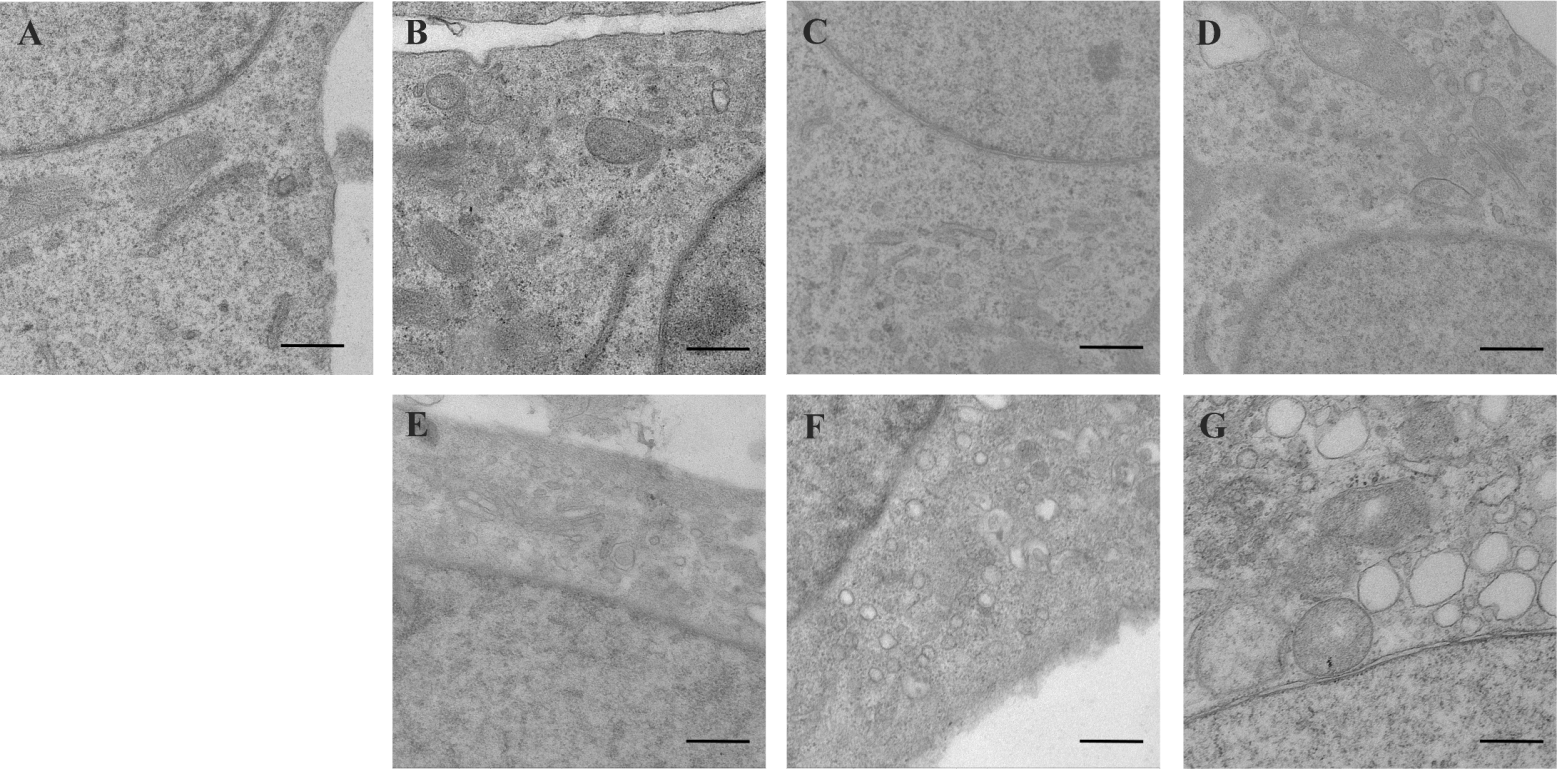
Progression of alterations in PRRSV-infected cells. Electron micrographs showing fixed PRRSV-infected cells versus mock (A) at 4 h (B), 8 h (C), 12 h (D), 16 h (E), 24 h (F), and 36 h (G). A minimum of 100 cells was detected for morphological alterations that appear during PRRSV invasion. An image of each typical area is represented. Scale bars, 500 nm.

To identify PRRSV-induced changes to intracellular membranes at the ultrastructural level, a timeline study was conducted to visualize virus-induced membrane alterations during infection. In contrast to mock-infected cells (Fig 3A), alterations to intracellular membranes were rarely detected in PRRSV-infected cells during the early phase (4–12 h.p.i) (Fig 3B, 3C and 3D). Vesicles appeared at 16 h.p.i., and more MVs appeared in the cytoplasm and became larger in size at 24 h.p.i. (Fig 3E). At 36 h.p.i., most membrane vesicles were empty and occupied large amounts of space in infected cells (Fig 3F). These results demonstrated that membrane vesicles were coupled with increasing viral RNA replication, suggesting that the formation of these vesicles may contribute to viral metabolism.

### ER and autophagosomes were involved in viral replication and assembly

**Fig 4:**
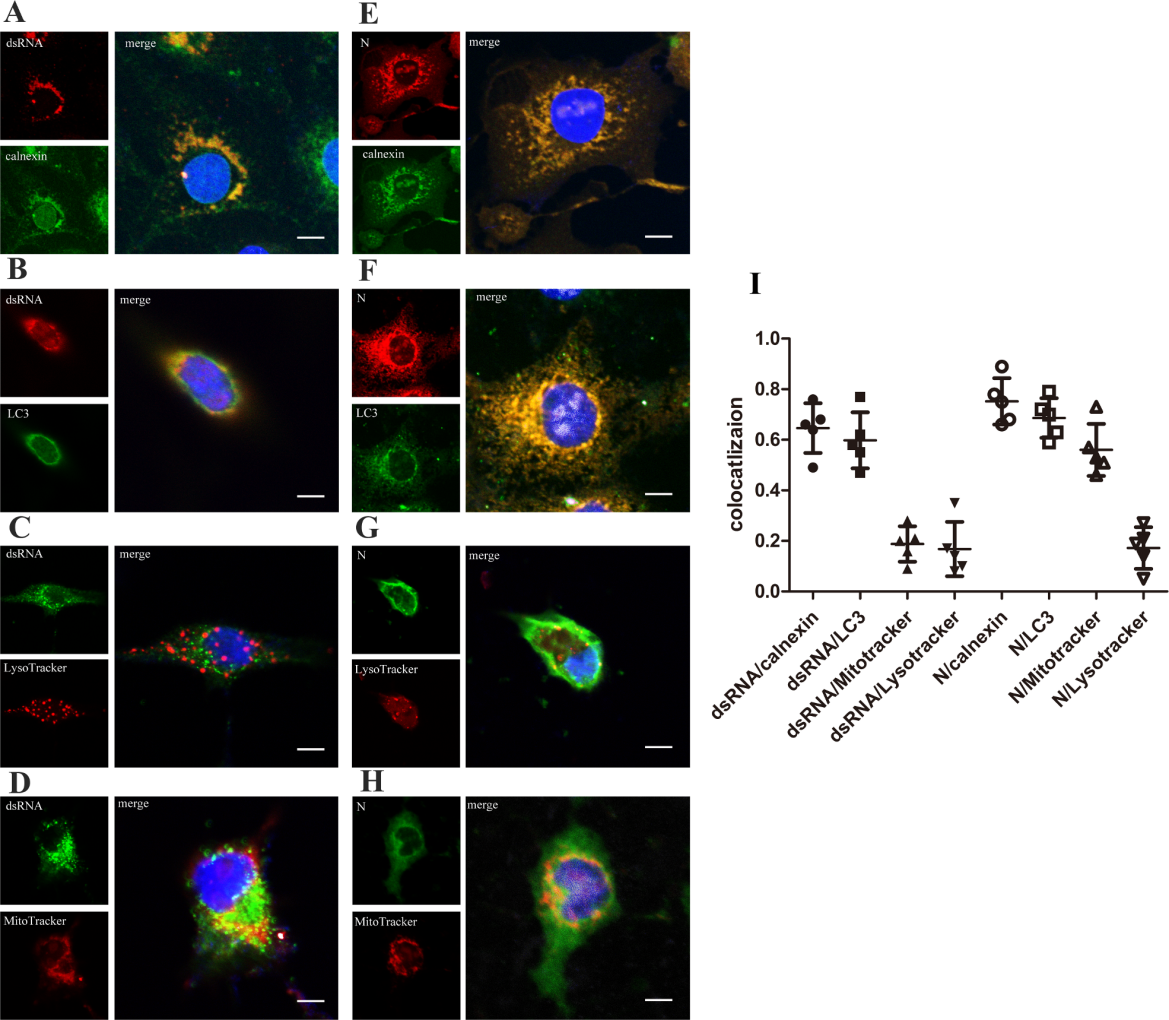
Localization of the viral N protein and dsRNA in Marc-145 cells infected with PRRSV. At the 24 h time point, Marc-145 cells were fixed and performed for immunofluorescence as mentioned in the Materials and Methods after PRRSV infection at an MOI of 1. Images of samples were visualized using a Nikon TE2000-E inverted confocal microscope at 63× magnification. (A)—(H) The cellular nuclei were dyed with DAPI (blue). Cells were co-stained with the J2 monoclonal anti-dsRNA antibody and (A) the anti-calnexin monoclonal antibody, (B) the anti-LC3 monoclonal antibody, (C) LysoTracker Red or (D) MitoTracker Red. Cells were costained with the monoclonal anti-NS1 antibody and (E) the anti-calnexin monoclonal antibody, (F) the anti-LC3 monoclonal antibody, (G) LysoTracker Red or (H) MitoTracker Red. Scale bars, 10 μm. (I) The graph showing Pearson’s correlation coefficients for each markers in (A)~(H), values above 0.5 were considered as colocalization.

To determine which organelles are involved in PRRSV replication, immunofluorescence microscopy was used to analyse PRRSV-infected Marc-145 cells. PRRSV is a positive-strand RNA virus that generates negative-strand RNA during propagation and transcription, and this negative-strand RNA is employed as a template for the assembly of positive-strand RNA. This process produces dsRNA intermediates, and dsRNA detection in virus-infected cells reflects viral replication. Cells contain many organelles; the ER is the main site involved in protein synthesis, and the autophagosome is involved in the phagocytic cell’s own metabolism and invading molecules or viruses. Mitochondrial metabolism provides energy for cells, and lysosomes are responsible for the decomposition of certain types of metabolized organic matter. In mock-infected cells, low amounts of dsRNA or N protein were immunolabeled (S1 Fig). At 24 h.p.i., Marc-145 cells infected with PRRSV were fixed, and PRRSV dsRNA, N protein and cellular proteins were detected using mono-specific antibodies, antibodies (calnexin, LC3) and organelle markers (MitoTracker, LysoTracker), respectively. To better explain the colocalization of two markers, we quantified the overlap coefficient using Pearson’s correlation, and values above 0.5 indicated colocalization. PRRSV dsRNA and N protein were mainly located outside of the nucleus and colocalized with calnexin or LC3 (Fig 4A, 4B, 4E, 4F and 4I), with Pearson’s correlation values of 0.65±0.10, 0.60±0.11, 0.75±0.09 and 0.68±0.08, respectively. Additionally, colocalization was detected between N protein and MitoTracker RED, which is a marker of mitochondria (Fig 4H and 4I), with a Pearson’s correlation value of 0.56±0.10; however, PRRSV dsRNA and MitoTracker RED demonstrated little evidence of colocalization (0.19±0.07) (Fig 4D and 4I). Neither PRRSV protein N nor dsRNA colocalized with lysosomes stained with LysoTracker (0.17±0.08 and 0.17±0.10) (Fig 4C, 4G and 4I). In conclusion, the ER and autophagosomes were the sites of viral replication and assembly, while some virus particles may have attached to the mitochondria.

### PRRSV RNA synthesis and assembly were exclusively associated with the ER

**Fig 5:**
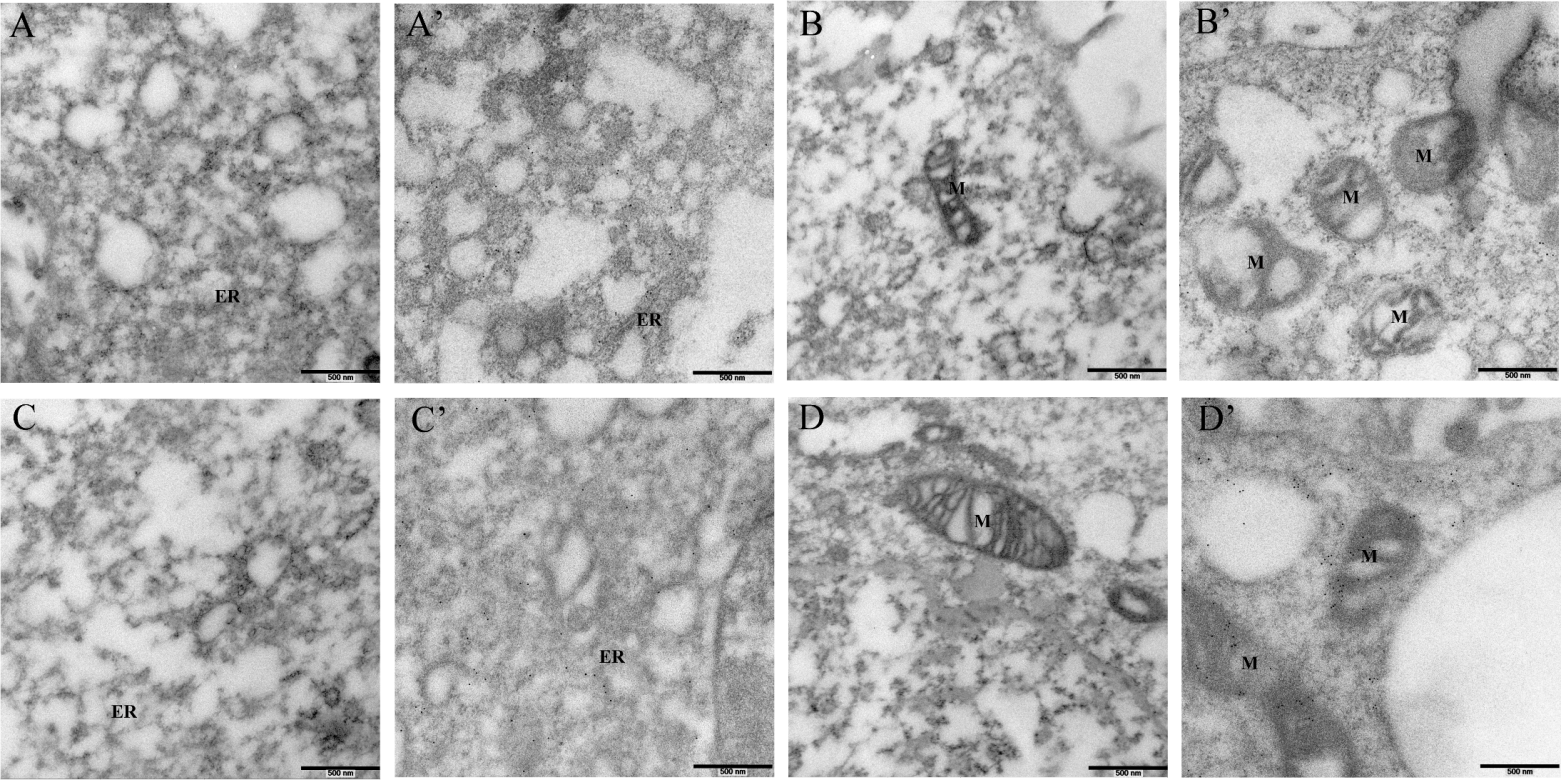
IEM of PRRSV-infected cells. PRRSV at an MOI of 1 was transfected into Marc-145 cells, and at 24hours post-infection, cells were immobilized, dehydrated, and inserted as described in the Materials and Methods(A’, B’, C’ and D’). Mock infected Marc-145 cells served as controls (A, B, C and D), Ultrathin sections were marked with antibodies against J2 (A, A’, B and B’) and N protein (C, C’ D and D’). All of the gold particles labeled the cytoplasm of infected cells. Anti-dsRNA labeling was mostly associated with ER and MV (A’), and few gold particles were observed in the mitochondria (M) (B’). Anti-N antibodies displayed labeling on the ER and mitochondria in infected cells (C’ and D’).

To confirm the localization of PRRSV dsRNA and N protein in the cellular ultrastructure, we used IEM to observe infected cells. In previous studies, EAV replicated its RNA genome on membrane structures originating from the ER [28]. In this study, to analyse the RNA derived from the viral duplication process, dsRNA was labelled with immunogold. Compared with mock-infected cells (Fig 5A), dsRNA coated with gold particles in PRRSV-infected cells was almost uniquely associated with vesicles or the ER (Fig 5A’) but was not significantly correlated with other organelles, such as the mitochondria (Fig 5B and 5B’). Similarly, we used the IEM method to gold-label the viral structural N protein. PRRSV-induced vesicles were apparent, and immunogold labelling revealed vesicles containing the N protein of PRRSV (Fig 5C and 5C’). Some N protein was also associated with mitochondria (Fig 5D and 5D’). In addition, we observed gold particles in the cytoplasm. Taken together, our results demonstrate that PRRSV-induced vesicles contain large amounts of dsRNA and N protein, which is instrumental for virus replication and synthesis. In contrast, the mitochondria maybe only serve as a temporary docking site for N protein.

### Analysis of the changes in membrane structures induced by PRRSV using ET

**Fig 6:**
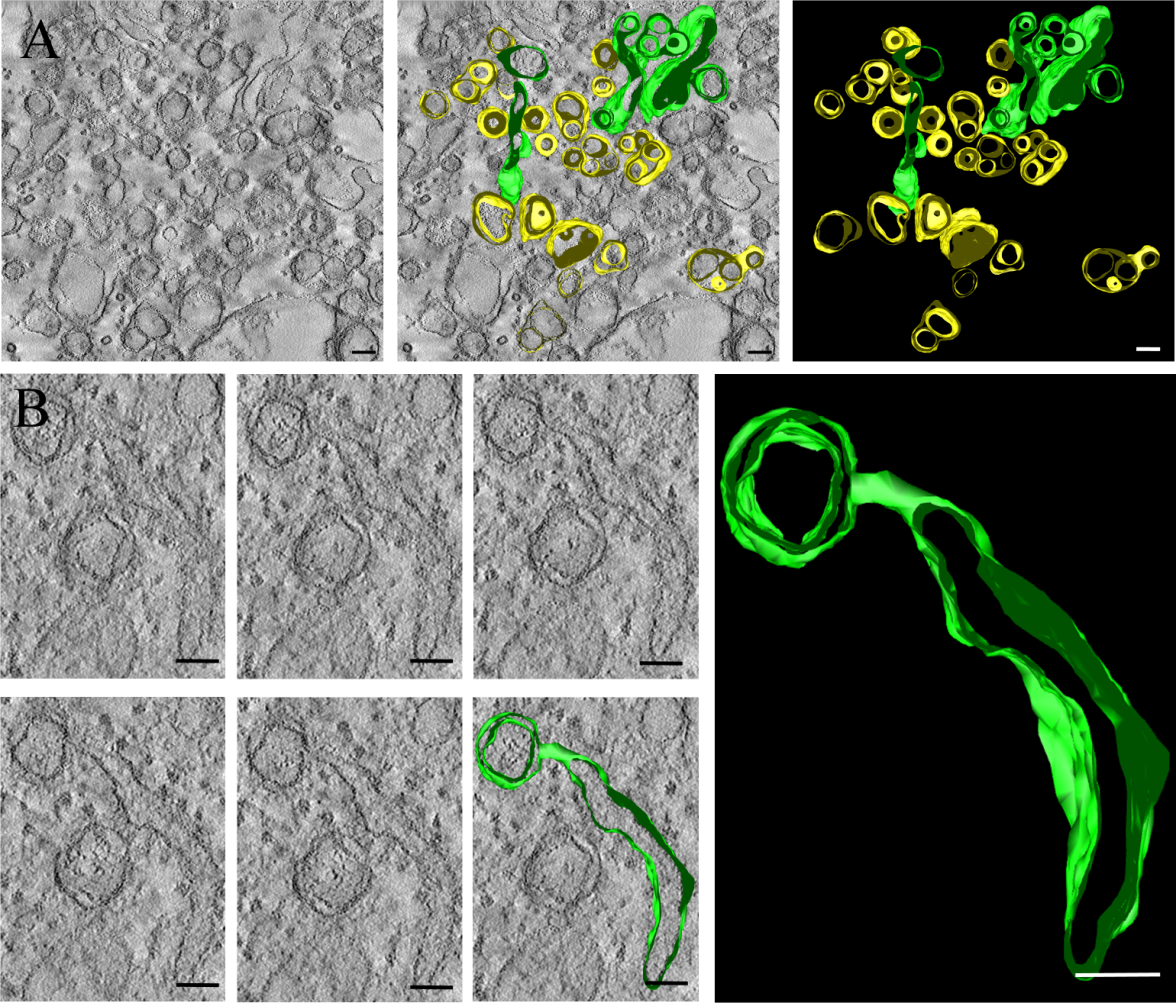
ET Reveals the RVN of interconnected ER-derived DMVs. Marc-145 cells were infected with PRRSV at an MOI of 1, fixed at 24 h.p.i, and processed for ET as mentioned in the Materials and Methods. (A) Left: slice of a dual-axis tomogram presenting the various membrane changes; right, 3D structure of the integrated tomogram. ER membranes and vesicles interconnected with ER lumens are described in light green; PRRSV-induced vesicles are depicted in light yellow. Scale bars, 100 nm (B) Left: slice of a dual-axis tomogram showing that a DMV is linked to the ER lumen; middle and right: this tomogram and the 3D membrane rendering of the DMVs structure are shown. These tomograms are shown in S1 Movie and S2 Movie. Scale bars, 50nm.

To understand the alterations in membrane structure induced by PRRSV, ET analysis was performed on 300-nm sections of Marc-145 cells 24 h after infection with PRRSV. As shown, some DMVs were continuous with the ER lumen. PRRSV-induced DMVs appeared in exvaginations, which were connected to the ER lumen through a short-necked structure (Fig 6A). Three-dimensional reconstruction revealed that the shapes of these structures were anomalous. Some were branched tubular structures (Fig 6B and S1 Movie), exemplified by a continuous snapshot and a 3D surface scenograph of tomograms obtained from cells analysed at 24 h.p.i. (Fig 6B and S2 Movie). The association of DMVs with the ER through their outer membranes suggested that these interactions may depict the intermediate stages of DMV formation, before DMVs are isolated from the ER. Indeed, many DMVs were tightly connected to the ER lumen with visible neck-like structures, whereas few DMVs were not connected to the ER lumen at all. These findings demonstrate that DMVs are isolated from the ER.

### PRRSV assembly occurs in RVN

**Fig 7:**
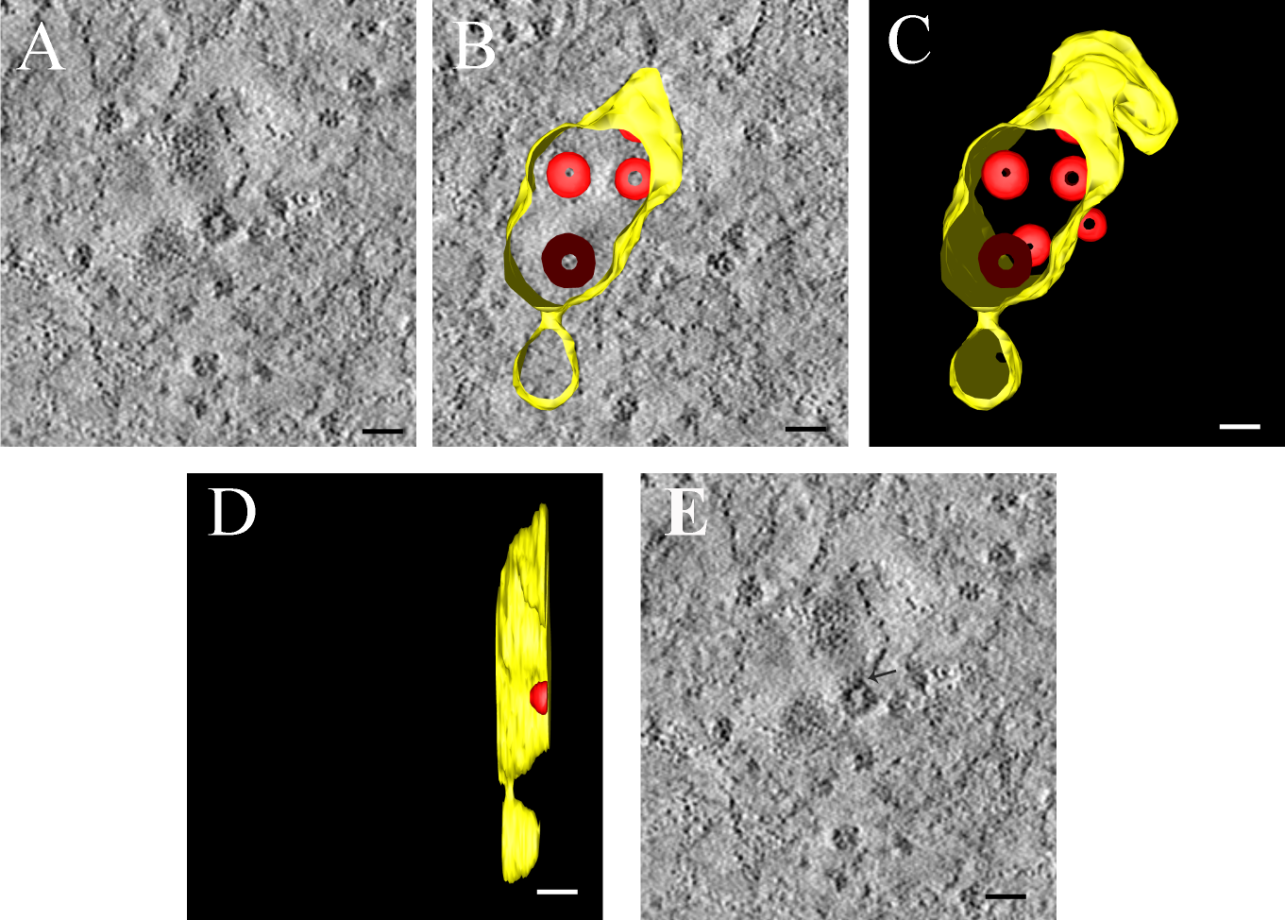
ET manifests the relationship between MVs and virus particles derived from ER membranes and captures virus budding. Marc-145 cells were infected with PRRSV at an MOI of 1, fixed at 24 hpi, and processed for ET as described in the Materials and Methods. (A) Close inspection of an ET ultra-thin slice displays an association between membrane vesicles and ER lumen-containing virus particles, with viral particles assembling at the limiting membrane of the vesicle. (B) The 3D structure model of the virus-induced structures in (A) depicts the relationship between membrane vesicles and the membranes enclosing the virus particles. The membrane vesicle is depicted in yellow and virus particles in red. (C) Final 3D surface-rendered model showing the connection between the viral particle and the ER derived membrane vesicle. (D) A side view of the 3D model represents the connection between virus budding and the membrane vesicle. This tomogram is shown in S3 Movie. (E) The particle was connected with the membrane which wrapped the particles, the arrow is the connection.

Subsequently, we found that PRRSV particles were encapsulated in infected Marc-145 cells using ET, but we did not find any evidence of virus particles in bi-dimensional normal sections at 100 nm. The ER enveloped a number of viral particles (Fig 7A, 7B and 7C and S3 Movie), and the ER was also connected with virus-induced vesicles through short neck structures, thus constituting an RVN and indicating the synergistic functions of these two structures. The vesicles were clearly induced to form an RVN, but the mechanism of vesicle formation and the role of PRRSV in their formation, as deduced for coronavirus [20], have not been fully elucidated.

Our previous experiments detected staining for anti-dsRNA or anti-N antibodies using IEM; however, the antibody concentrations were too low to detect specific staining. By using ET, we observed that virus particles and the ER are connected (Fig 7E); therefore, viral-modified membranes and the RVN produced by PRRSV-infected cells were inextricably linked to viral anabolism. After infection, an RNA-nucleocapsid protein complex was formed and attached to the membrane, allowing for the efficient assembly, production and delivery of viral RNA from PRRSV progeny. We used ET to study the site of virus budding and revealed that the virus particles are located in an RVN consisting of the ER and membrane vesicles; the particles are also connected the outer membrane of the ER.

## Discussion

The requirement for organelles and the involvement of intracellular modules in PRRSV replication have long been controversial. Over the decades, some EM studies have revealed relationships between the endomembrane and viral replication [29–31]. Previous studies investigating the replication structure of PRRSV reported that progeny virions accumulate in the intimal compartment of the cell and are released into the extracellular space via exocytosis [32]. Analysis of ultrastructural membrane structure changes in infected cells is an important approach to understand how RNA viruses modify cellular membrane structures to create an environment for their genomic replication and gene expression.

Ultrastructural research defines a framework of space and time that integrates cell biology and biochemistry data from infected systems to help us understand the cycle of RNA virus replication [4, 9]. In this work, we combined ET, IEM and a number of molecular biology tools to generate a model of PRRSV-infected cells. Our results confirmed that the DMV structures in Marc-145 cells are induced by PRRSV; these DMV structures are similar to the RVN structures induced by EAV. However, the vesicles were notably smaller than those produced by coronaviruses.

Previous transmission EM studies in EAV-infected cells revealed that cellular membrane rearrangement is reduced by virus infection [33]. Using Marc-145 cells as a model, our study demonstrates a correlation between functional membrane remodelling and RNA synthesis during PRRSV replication (Fig 1). We comprehensively monitored membrane rearrangements and viral replication every 4 h from 0 to 16 h.p.i. and every 12 h after 24 h.p.i. DMVs appeared at 16 h.p.i. and became larger at 24 h.p.i.; during this period, levels of newly synthesized viral RNA began to increase. These results imply a link between membrane remodelling and RNA synthesis. However, at 36 h.p.i., the size and number of DMVs were not significantly different, and large numbers of vacuolar structures appeared (Fig 3). We inferred that the decreased viral RNA synthesis rate we observed was mainly attributable to increased viral genome packaging in the cytoplasm, resulting in an increased rate of viral particle release, thus preventing RNA accumulation. This may be induced by the consumption of RNA produced by the cells.

After PRRSV infection, cells appear to engage in vesicle trafficking and synthesis to provide sites for viral RNA translation, replication and viral assembly [7]. Although the role of vesicles as RNA replication sites has been established, the hypothesis stating that PRRSV RNA synthesis occurs in the vesicle lumen requires further exploration through staining for dsRNA markers. The formation of RTCs is induced by positive-strand RNA viruses during infection; these RTCs contain viral proteins, host proteins, viral nucleic acids, and the host ER membrane and dsRNA formed during virus replication. Autophagy has been shown to be crucial for the replication of several positive-strand RNA viruses, such as poliovirus [34], coronavirus [35] and dengue virus [36]. Using immunofluorescence and IEM, we found that dsRNA synthesized by the virus localized on the ER and autophagosomes near the nucleus, indicating that the ER and autophagosomes are essential for viral replication (Figs 4 and 5). In our study, the PRRSV N protein co-localized with mitochondria, suggesting that, unlike previous reports of mitophagy [6], the viral capsid protein was attached to the internal network. Morphological changes in the mitochondrial membrane compared with uninfected cells have not yet been detected.

Using EM, we detected cell membrane rearrangements induced by PRRSV. These structures are typically shown as vesicles, including single-membrane vesicles and DMVs. In our experiments, 100-nm-thick samples were used to observe the shapes of the structures in PRRSV-infected cells. Large numbers of DMVs were easily observed after infection, mostly around the ER (Fig 2). However, two-dimensional transmission EM only showed cross-sections of these vesicles. Some organelles secreted by the capsule were difficult to see in previous reports, and our data show that some information was misleading.

Previous studies have shown that PRRSV invasion remarkably increases the number of single-membrane vesicles or DMVs in the cytoplasm [37]. Many positive-strand RNA virus-induced structures are composed of DMVs [30]. West Nile virus stimulates retraction of the ER membrane, which links to the cytosol via small pores, modulating the release of viral RNA [14]. Relevant findings were confirmed with dengue virus, another flavivirus. Polioviruses demonstrate extensive membrane recombination derived from sections of an anterograde membrane trafficking system to produce single-membrane vesicles and DMVs and form complex network structures [16]. Data from other studies have shown that the propagation of positive-stranded RNA viruses is connected to intracellular membranes; virus-induced membrane alterations are connected with viral RNA replication, including genome replication, and involve changes in the mitochondria and Golgi [9–11, 38]. Plant pathogens such as BBSV induce widespread rearrangement of the ER to form dotted spots or plaques in the cytoplasm, and tomato bushy stunt virus induces more peroxisomal and chloroplast remodelling [39]. In our research, PRRSV infection produced large numbers of vesicles, most of which were connected with the ER membrane structure and part of the constructed RVNs (Fig 6). Notably, DMVs contained a channel linking their exterior to the ER. The diameter of this channel, which connected PRRSV-induced vesicles with the cytoplasm, must be sufficiently large for the exchange of RNA products and nucleotides with cytoplasm. This result is consistent with our hypothesis that this channel provides the required components for viral RNA synthesis sites. Our results suggest that the pattern of cellular pathology produced by PRRSV infection is similar to those of HCV and SARS-CoV and results in large numbers of complex structures that affect the reorganization of cellular membrane structures.

In addition to identifying vesicles linked to the ER, we also found ER-enveloped virus particles in PRRSV-infected cells. It has been reported that pre-assembled nucleocapsid budding occurs on intracellular membranes [40]. Some previous studies provided particularly interesting EM observations from low-temperature electrophysiological experiments showing the structure of PRRSV particles extending from the cytosol to the budding process [41]. The core was described as a double-layered hollow structure with an average diameter of 39 nm. ET was first used to demonstrate the 3D resolution of the FHV replication complex [42]. This technique has since been applied to characterize the 3D characteristics of a variety of positive-strand RNA viruses, including Picornaviridae viruses, Flaviviridae viruses, hepatitis C virus and Coronaviridae viruses [10, 43]. In this study, ET captured viral particles budding from the ER membrane (Fig 7). Viral particles are synthesized in vesicles derived from the ER and are secreted into the cytoplasm to exert their viral activities [19]. These structures have previously been detected exclusively in flavivirus [31] and coronavirus-infected cells. These similar results imply that certain positive-strand RNA viruses accomplish their replication and assembly by inducing the formation of replication complexes [44].

Overall, our results demonstrate similar structures produced by membrane rearrangement following PRRSV infection and EAV infection. However, we identified some differences between membrane structures caused by PRRSV infection and other related viruses as well as novel membrane structures. These membrane structures are similar to the RNA synthesis sites confirmed for other positive single-stranded RNA viruses. More importantly, for the first time, we used ET to reconstitute nidovirus particle synthesis sites. Further research should investigate whether vesicles provide sites for PRRSV RNA synthesis and determine which viral proteins induce membrane rearrangement. To our knowledge, this study is the first to present the PRRSV-induced membrane changes associated with viral RNA replication in 3D models.

## Conclusion

In this study, we created a 3D reconstruction of the membrane rearrangement induced by PRRSV. Our findings provide highly valuable information regarding the biological context of PRRSV and show that PRRSV assists in the recruitment of membrane structures and related proteins to vesicle-associated processes. By using IEM and other molecular virology approaches, our results elucidate connections between PRRSV duplication, assembly and the course of virus-induced membrane remodelling. The findings of this study are valuable for understanding the life cycle of PRRSV-invaded cells in vitro.

## Supporting information

S1 Fig Localization of the viral N protein and dsRNA in mock-infected cells. Cells cultured on plates were fixed and processed for immunofluorescence as described in the Materials and Methods, and nuclei were stained with DAPI. DsRNA and protein N are stained in green. Scale bars, 10 μm.
(TIF)

S1 Movie Single-axis electron microscopy tomogram reconstructed from an ~300-nm-thick section of PRRSV-infected Marc-145 cells fixed at 24 h p.i. (corresponding to Fig 6). The animation according to the tomogram displays a large number of vesicles; among these, independent vesicle structures are depicted in yellow, and vesicles interconnected to the endoplasmic reticulum are depicted in green.
(MOV)

S2 Movie Single-axis electron microscopy tomogram reconstructed from an ~300-nm-thick section of PRRSV-infected Marc-145 cells fixed at 24 h p.i. (corresponding to Fig 6). The animation according to the tomogram shows that the ER is connected to the lateral side of a vesicle, suggesting that vesicles are secreted by the ER.
(MOV)

S3 Movie Single-axis electron microscopy tomogram reconstructed from an ~300-nm-thick section of PRRSV-infected Marc-145 cells fixed at 24 h p.i. (corresponding to Fig 7). The animation according to the tomogram shows that most virus particles were observed within the vesicle and that one particle was budding into the outside of the membrane vesicle, suggesting that these vesicles are the locations of PRRSV particle assembly and secretion.
(MOV)

## Abbreviations

3D: three-dimensional
DMV: double membrane vesicles
dsRNA: double-stranded RNA
EM: electron microscopy
ER: endoplasmic reticulum
ET: electron tomography
IEM: immune-electron microscopy
MV: membranous vesicles
PRRSV: porcine reproductive and respiratory syndrome virus
RVN: reticulovesicular network

## References

[1] Meulenberg JJ, Hulst MM, de Meijer EJ, Moonen PL, den Besten A, de Kluyver EP, et al Lelystad virus, the causative agent of porcine epidemic abortion and respiratory syndrome (PEARS), is related to LDV and EAV. Virology. 1993;192(1):62–72. 10.1006/viro.1993.1008 . 8517032 PMC7173055

[2] World Organization for Animal Health (OIE). World animal health in 2000. Part 1: reports on the animal health status and disease control methods and tables on incidence of list A diseases. World Organization for Animal Health (OIE), 2001.

[3] Misinzo G, Delputte PL, Meerts P, Drexler C, Nauwynck HJ. Efficacy of an inactivated PRRSV vaccine—Induction of virus-neutralizing antibodies and partial virological protection upon challenge In: Perlamn S, Holmes KV, editors. Nidoviruses: Toward Control of Sars and Other Nidovirus Diseases. Advances in Experimental Medicine and Biology 581 2006 p. 449–54. 10.1007/978-0-387-33012-9_81 PMC7122804 17037577

[4] Blanchard E, Roingeard P. Virus-induced double-membrane vesicles. Cellular Microbiology. 2015;17(1):45–50. 10.1111/cmi.12372 PubMed PMID: WOS:000346704300006. 25287059 PMC5640787

[5] Dea S, Sawyer N, Alain R, Athanassious R. Ultrastructural Characteristics and Morphogenesis of Porcine Reproductive and Respiratory Syndrome Virus Propagated in the Highly Permissive MARC-145 Cell Clone In: Talbot PJ, Levy GA, editors. Corona- and Related Viruses: Current Concepts in Molecular Biology and Pathogenesis. Boston, MA: Springer US; 1995 p. 95–8. 10.1007/978-1-4615-1899-0_13 8830552

[6] Li SF, Wang JX, Zhou A, Khan FA, Hu L, Zhang SJ. Porcine reproductive and respiratory syndrome virus triggers mitochondrial fission and mitophagy to attenuate apoptosis. Oncotarget. 2016;7(35):56002–12. 10.18632/oncotarget.10817 PubMed PMID: WOS:000386911600006. 27463011 PMC5302892

[7] Fang Y, Snijder EJ. The PRRSV replicase: Exploring the multifunctionality of an intriguing set of nonstructural proteins. Virus Research. 2010;154(1–2):61–76. 10.1016/j.virusres.2010.07.030 PubMed PMID: WOS:000285124600007. 20696193 PMC7114499

[8] Oudshoorn D, van der Hoeven B, Limpens R, Beugeling C, Snijder EJ, Barcena M, et al Antiviral innate immune response interferes with the formation of replication-associated membrane structures induced by a positive-strand RNA virus. MBio. 2016;7(6):e01991–16. doi: e01991 10.1128/mBio.01991-16 PubMed PMID: WOS:000392079500053. 27923923 PMC5142621

[9] Harak C, Lohmann V. Ultrastructure of the replication sites of positive-strand RNA viruses. Virology. 2015;479:418–33. 10.1016/j.virol.2015.02.029 PubMed PMID: WOS:000354909500037. 25746936 PMC7111692

[10] Paul D, Bartenschlager R. Architecture and biogenesis of plus-strand RNA virus replication factories. World J Virol. 2013;2(2):32–48. 10.5501/wjv.v2.i2.32 24175228 PMC3785047

[11] Denison MR. Seeking membranes: Positive-strand RNA virus replication complexes. PLoS Biol. 2008;6(10):2098–100. doi: e270 10.1371/journal.pbio.0060270 PubMed PMID: WOS:000260423900006. 18959488 PMC2573941

[12] Miller S, Krijnse-Locker J. Modification of intracellular membrane structures for virus replication. Nature Reviews Microbiology. 2008;6(5):363–74. 10.1038/nrmicro1890 PubMed PMID: WOS:000255099100012. 18414501 PMC7096853

[13] Urakova N, Strive T, Frese M. RNA-dependent RNA polymerases of both virulent and benign rabbit caliciviruses induce striking rearrangement of golgi membranes. PLoS One. 2017;12(1):e0169913. doi: e0169913 10.1371/journal.pone.0169913 PubMed PMID: WOS:000391844200057. 28072826 PMC5224886

[14] Gillespie LK, Hoenen A, Morgan G, Mackenzie JM. The endoplasmic reticulum provides the membrane platform for biogenesis of the flavivirus replication complex. J Virol. 2010;84(20):10438–47. 10.1128/JVI.00986-10 PubMed PMID: WOS:000282642600001. 20686019 PMC2950591

[15] Suarez C, Andres G, Kolovou A, Hoppe S, Salas ML, Walther P, et al African swine fever virus assembles a single membrane derived from rupture of the endoplasmic reticulum. Cell Microbiol. 2015;17(11):1683–98. 10.1111/cmi.12468 PubMed PMID: WOS:000363873400011. 26096327

[16] Belov GA, Nair V, Hansen BT, Hoyt FH, Fischer ER, Ehrenfeld E. Complex dynamic development of poliovirus membranous replication complexes. J Virol. 2012;86(1):302–12. 10.1128/JVI.05937-11 PubMed PMID: WOS:000298347700029. 22072780 PMC3255921

[17] Fontana J, Lopez-Montero N, Elliott RM, Fernandez JJ, Risco C. The unique architecture of Bunyamwera virus factories around the Golgi complex. Cell Microbiol. 2008;10(10):2012–28. 10.1111/j.1462-5822.2008.01184.x PubMed PMID: WOS:000259086900009. 18547336 PMC7162186

[18] Spuul P, Balistreri G, Kaariainen L, Ahola T. Phosphatidylinositol 3-kinase-, actin-, and microtubule-dependent transport of semliki forest virus replication complexes from the plasma membrane to modified lysosomes. J Virol. 2010;84(15):7543–57. 10.1128/JVI.00477-10 PubMed PMID: WOS:000279989800012. 20484502 PMC2897599

[19] Ulasli M, Verheije MH, de Haan CA, Reggiori F. Qualitative and quantitative ultrastructural analysis of the membrane rearrangements induced by coronavirus. Cell Microbiol. 2010;12(6):844–61. 10.1111/j.1462-5822.2010.01437.x 20088951 PMC7159092

[20] Knoops K, Kikkert M, van den Worm SHE, Zevenhoven-Dobbe JC, van der Meer Y, Koster AJ, et al SARS-coronavirus replication is supported by a reticulovesicular network of modified endoplasmic reticulum. Plos Biology. 2008;6(9):1957–74. doi: e226 10.1371/journal.pbio.0060226 PubMed PMID: WOS:000259783600018. 18798692 PMC2535663

[21] de Wilde AH, Raj VS, Oudshoorn D, Bestebroer TM, van Nieuwkoop S, Limpens RW, et al MERS-coronavirus replication induces severe in vitro cytopathology and is strongly inhibited by cyclosporin A or interferon-alpha treatment. J Gen Virol. 2013;94(Pt 8):1749–60. 10.1099/vir.0.052910-0 23620378 PMC3749523

[22] Maier HJ, Hawes PC, Cottam EM, Mantell J, Verkade P, Monaghan P, et al Infectious bronchitis virus generates spherules from zippered endoplasmic reticulum membranes. Mbio. 2013;4(5):00801–13. 10.1128/mBio.00801-13 PMC3812713 24149513

[23] Metwally S, Mohamed F, Faaberg K, Burrage T, Prarat M, Moran K, et al Pathogenicity and molecular characterization of emerging porcine reproductive and respiratory syndrome virus in Vietnam in 2007. Transbound Emerg Dis. 2010;57(5):315–29. 10.1111/j.1865-1682.2010.01152.x PubMed PMID: WOS:000281667300003. 20629970

[24] Zhao HJ, Ji QQ, Zhao GY, Song ZW, Du BZ, Nie Y, et al Damage of zona pellucida reduces the developmental potential and quality of porcine circovirus type 2-infected oocytes after parthenogenetic activation. Theriogenology. 2014;82(6):790–9. 10.1016/j.theriogenology.2014.06.003 PubMed PMID: WOS:000341542800002. 25062959

[25] Zhang QF, Cui JM, Huan XJ, Zheng HY, Huang JH, Ling F, et al The life cycle of SARS coronavirus in Vero E6 cells. J Med Virol. 2004;73(3):332–7. 10.1002/jmv.20095 PubMed PMID: WOS:000221677700002. 15170625 PMC7166737

[26] Zhu HL, Li HM, Wang P, Chen MK, Huang ZW, Li KP, et al Persistent and acute chlamydial infections induce different structural changes in the Golgi apparatus. Int J Med Microbiol. 2014;304(5–6):577–85. 10.1016/j.ijmm.2014.03.002 PubMed PMID: WOS:000339775600007. 24780199

[27] Molenkamp R, van Tol H, Rozier BCD, van der Meer Y, Spaan WJM, Snijder EJ. The arterivirus replicase is the only viral protein required for genome replication and subgenomic mRNA transcription. Journal of General Virology. 2000;81:2491–6. PubMed PMID: WOS:000089557000016. 10.1099/0022-1317-81-10-2491 10993938

[28] Pedersen KW, van der Meer Y, Roos N, Snijder EJ. Open reading frame 1a-encoded subunits of the arterivirus replicase induce endoplasmic reticulum-derived double-membrane vesicles which carry the viral replication complex. J Virol. 1999;73(3):2016–26. PubMed PMID: WOS:000078603800032. 9971782 10.1128/jvi.73.3.2016-2026.1999 PMC104444

[29] Peng L, Ryazantsev S, Sun R, Zhou ZH. Three-Dimensional Visualization of Gammaherpesvirus Life Cycle in Host Cells by Electron Tomography. Structure. 2010;18(1):47–58. 10.1016/j.str.2009.10.017 PubMed PMID: WOS:000273859700009. 20152152 PMC2866045

[30] Romero-Brey I, Merz A, Chiramel A, Lee JY, Chlanda P, Haselman U, et al Three-dimensional architecture and biogenesis of membrane structures associated with hepatitis C virus replication. PLoS Pathog. 2012;8(12):e1003056. doi: e1003056 10.1371/journal.ppat.1003056 PubMed PMID: WOS:000312907100015. 23236278 PMC3516559

[31] Welsch S, Miller S, Romero-Brey I, Merz A, Bleck CKE, Walther P, et al Composition and three-dimensional architecture of the dengue virus replication and assembly sites. Cell Host Microbe. 2009;5(4):365–75. 10.1016/j.chom.2009.03.007 PubMed PMID: WOS:000265571600009. 19380115 PMC7103389

[32] Dea S, Sawyer N, Alain R, Athanassious R. Ultrastructural characteristics and morphogenesis of porcine reproductive and respiratory syndrome virus propagated in the highly permissive MARC-145 cell clone. Adv Exp Med Biol. 1995;380:95–8. . 8830552 10.1007/978-1-4615-1899-0_13

[33] Knoops K, Barcena M, Limpens R, Koster AJ, Mommaas AM, Snijder EJ. Ultrastructural Characterization of Arterivirus Replication Structures: Reshaping the Endoplasmic Reticulum To Accommodate Viral RNA Synthesis. Journal of Virology. 2012;86(5):2474–87. 10.1128/JVI.06677-11 PubMed PMID: WOS:000300536800008. 22190716 PMC3302280

[34] Jackson WT, Giddings TH, Taylor MP, Mulinyawe S, Rabinovitch M, Kopito RR, et al Subversion of cellular autophagosomal machinery by RNA viruses. PLoS Biol. 2005;3(5):861–71. doi: e156 10.1371/journal.pbio.0030156 PubMed PMID: WOS:000229125400014. 15884975 PMC1084330

[35] Prentice E, Jerome WG, Yoshimori T, Mizushima N, Denison MR. Coronavirus replication complex formation utilizes components of cellular autophagy. J Biol Chem. 2004;279(11):10136–41. 10.1074/jbc.M306124200 PubMed PMID: WOS:000220050400059. 14699140 PMC7957857

[36] Lee YR, Lei HY, Liu MT, Wang JR, Chen SH, Jiang-Shieh YF, et al Autophagic machinery activated by dengue virus enhances virus replication. Virology. 2008;374(2):240–8. 10.1016/j.virol.2008.02.016 PubMed PMID: WOS:000255735900003. 18353420 PMC7103294

[37] Yoo D, Wootton SK, Li G, Song C, Rowland RR. Colocalization and interaction of the porcine arterivirus nucleocapsid protein with the small nucleolar RNA-associated protein fibrillarin. J Virol. 2003;77(22):12173–83. 10.1128/JVI.77.22.12173-12183.2003 PubMed PMID: WOS:000186294300032. 14581554 PMC254285

[38] Blanchard E, Roingeard P. Virus-induced double-membrane vesicles. Cell Microbiol. 2015;17(1):45–50. 10.1111/cmi.12372 PubMed PMID: WOS:000346704300006. 25287059 PMC5640787

[39] McCartney AW, Greenwood JS, Fabian MR, White KA, Mullen RT. Localization of the tomato bushy stunt virus replication protein p33 reveals a peroxisome-to-endoplasmic reticulum sorting pathway. Plant Cell. 2005;17(12):3513–31. 10.1105/tpc.105.036350 PubMed PMID: WOS:000233655700023. 16284309 PMC1315385

[40] Junjhon J, Pennington JG, Edwards TJ, Perera R, Lanman J, Kuhn RJ. Ultrastructural Characterization and Three-Dimensional Architecture of Replication Sites in Dengue Virus-Infected Mosquito Cells. Journal of Virology. 2014;88(9):4687–97. 10.1128/JVI.00118-14 PubMed PMID: WOS:000334353900008. 24522909 PMC3993787

[41] Spilman MS, Welbon C, Nelson E, Dokland T. Cryo-electron tomography of porcine reproductive and respiratory syndrome virus: organization of the nucleocapsid. J Gen Virol. 2009;90(Pt 3):527–35. 10.1099/vir.0.007674-0 19218197

[42] Kopek BG, Perkins G, Miller DJ, Ellisman MH, Ahlquist P. Three-dimensional analysis of a viral RNA replication complex reveals a virus-induced mini-organelle. Plos Biology. 2007;5(9):2022–34. doi: e220 10.1371/journal.pbio.0050220 PubMed PMID: WOS:000249552300020. 17696647 PMC1945040

[43] Hsu N-Y, Ilnytska O, Belov G, Santiana M, Chen Y-H, Takvorian PM, et al Viral Reorganization of the Secretory Pathway Generates Distinct Organelles for RNA Replication. Cell. 2010;141(5):799–811. 10.1016/j.cell.2010.03.050 PubMed PMID: WOS:000278132900015. 20510927 PMC2982146

[44] Ulasli M, Verheije MH, de Haan CAM, Reggiori F. Qualitative and quantitative ultrastructural analysis of the membrane rearrangements induced by coronavirus. Cell Microbiol. 2010;12(6):844–61. 10.1111/j.1462-5822.2010.01437.x PubMed PMID: WOS:000277381700011. 20088951 PMC7159092

