# Supplementary figures and images for: An integrated analysis of membrane remodeling during porcine reproductive and respiratory syndrome virus replication and assembly
Source: PLoS One. 2018 Jul 24;13(7):e0200919. doi: 10.1371/journal.pone.0200919 (PMC6057628; doi:10.1371/journal.pone.0200919)

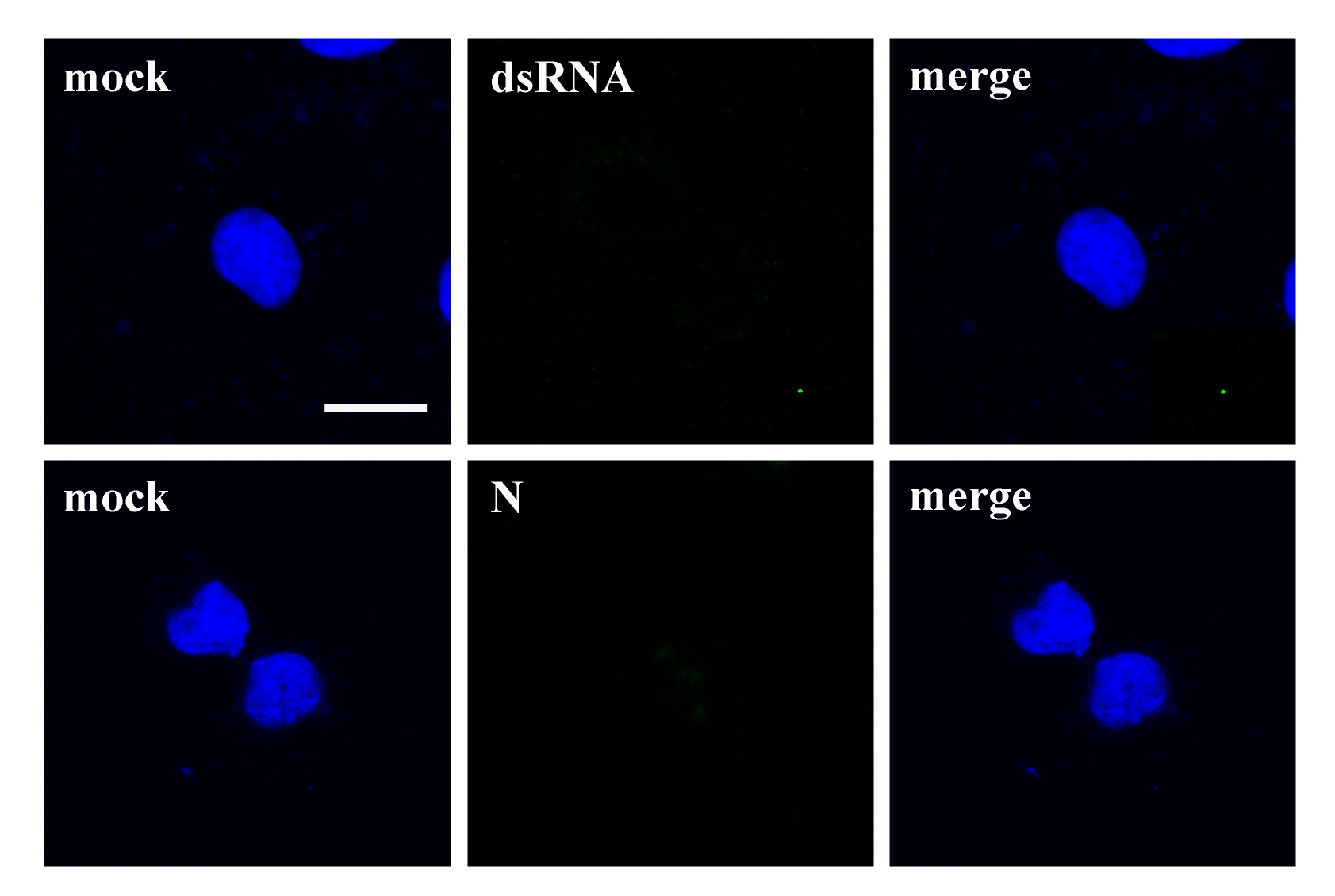

Supplement: S1 Fig — Cells cultured on plates were fixed and processed for immunofluorescence as described in the Materials and Methods, and nuclei were stained with DAPI. DsRNA and protein N are stained in green. Scale bars, 10 μm. (TIF) [file pone.0200919.s001.tif]
